# Supplementary material for: Genome-wide CRISPR Screens in T Helper Cells Reveal Pervasive Crosstalk between Activation and Differentiation
Source: Cell. 2019 Feb 7;176(4):882–896.e18. doi: 10.1016/j.cell.2018.11.044 (PMC6370901; doi:10.1016/j.cell.2018.11.044)
Supplement: Data S2. Processed Data from All the Steps of the Analysis, Related to Figure 1 [file mmc2.zip › supplemental data/motif analysis/Stat6_SRX021632_homer/homerResults/motif15.similar.html]

motif15

## Information for motif15

T
C
A
G
C
T
G
A
C
T
G
A
G
C
T
A
T
A
C
G
G
C
A
T
T
C
A
G
C
G
T
A
C
T
G
A
G
C
T
A
T
A
C
G
G
A
C
T
  
Reverse Opposite:  

C
T
G
A
A
T
G
C
C
A
G
T
A
G
C
T
G
C
A
T
A
G
T
C
C
G
T
A
A
T
G
C
C
G
A
T
A
G
C
T
G
A
C
T
A
G
T
C
  

|  |  |
| --- | --- |
| p-value: | 1e-107 |
| log p-value: | -2.483e+02 |
| Information Content per bp: | 1.668 |
| Number of Target Sequences with motif | 720.0 |
| Percentage of Target Sequences with motif | 2.85% |
| Number of Background Sequences with motif | 268.0 |
| Percentage of Background Sequences with motif | 1.11% |
| Average Position of motif in Targets | 100.6 +/- 52.6bp |
| Average Position of motif in Background | 105.3 +/- 66.9bp |
| Strand Bias (log2 ratio + to - strand density) | -0.0 |
| Multiplicity (# of sites on avg that occur together) | 1.05 |
| Motif File: | file (matrix) reverse opposite |

### Similar de novo motifs found

|  |  |  |  |  |  |  |  |
| --- | --- | --- | --- | --- | --- | --- | --- |
| Rank | Match Score | Redundant Motif | P-value | log P-value | % of Targets | % of Background | Motif file |
| 1 | 0.928 | C G A T G C A T A G C T A G T C C T G A A C T G A C G T C G A T G A C T A G T C | 1e-92 | -213.773557 | 2.07% | 0.73% | motif file (matrix) |
| 2 | 0.881 | C G T A C G T A A T G C A C G T A C T G C T G A G T C A C G T A | 1e-91 | -211.051696 | 7.38% | 4.49% | motif file (matrix) |
| 3 | 0.754 | A T C G G T C A C G T A T C G A C G T A A T G C C G A T A T C G C T G A C G T A C G T A C G T A | 1e-47 | -109.547039 | 0.24% | 0.02% | motif file (matrix) |
| 4 | 0.677 | C G T A C G T A C G T A C G T A A C G T A C T G C G T A A C T G C G T A C G T A | 1e-15 | -35.741613 | 0.27% | 0.08% | motif file (matrix) |
